# Terpenoids Commonly Found in *Cannabis sativa* Do Not Modulate the Actions of Phytocannabinoids or Endocannabinoids on TRPA1 and TRPV1 Channels

**DOI:** 10.1089/can.2019.0099

**Published:** 2020-12-15

**Authors:** Marika Heblinski, Marina Santiago, Charlotte Fletcher, Jordyn Stuart, Mark Connor, Iain S. McGregor, Jonathon C. Arnold

**Affiliations:** ^1^ The Lambert Initiative for Cannabinoid Therapeutics, Brain and Mind Centre, The University of Sydney, Sydney, Australia.; ^2^ Faculty of Medicine and Health and School of Medical Sciences, The University of Sydney, Sydney, Australia.; ^3^ Faculty of Medicine and Health Sciences, Macquarie University, Macquarie Park, Sydney, Australia.; ^4^ Faculty of Science and School of Psychology, The University of Sydney, Sydney, Australia.

**Keywords:** terpenoid, TRPA1, TRPV1, phytocannabinoid, endocannabinoid, entourage effect

## Abstract

**Introduction:** *Cannabis sativa* produces hundreds of bioactive compounds, including cannabinoids and terpenoids. It has been proposed that cannabinoids act in synergy with terpenoids to produce the entourage effect, a concept used to explain the therapeutic benefits of medicinal cannabis. One molecular explanation for the entourage effect is that the terpenoids augment the actions of cannabinoids at their molecular drug targets in cells. We recently reported that terpenoids commonly found in cannabis do not influence the functional effects of Δ^9^-tetrahydrocannabinol (Δ^9^-THC) on cannabinoid 1 and cannabinoid 2 receptors. The present study aimed to extend on this research by examining whether terpenoids influence the effects of phytocannabinoids and endocannabinoids on human transient receptor potential ankyrin 1 (hTRPA1) and human transient receptor potential vanilloid 1 (hTRPV1) channels heterologously expressed in mammalian cells.

**Materials and Methods:** The activity of terpenoids, phytocannabinoids, and endocannabinoids was assessed in inducible HEK Flp-In T-Rex cells transfected with hTRPA1 and hTRPV1 channels, respectively. Real-time changes in intracellular calcium ([Ca]_i_) were measured using the Calcium 5 dye and a FlexStation 3 plate reader.

**Results:** α-pinene, β-pinene, β-caryophyllene, linalool, limonene, β-myrcene or α-humulene did not affect [Ca]_i_ in hTRPA1 and hTRPV1 overexpressing cells. Cinnamaldehyde (CA), Δ^9^-THC, and 2-arachidonoylglycerol (2-AG) activated TRPA1 receptors with high efficacy and similar potency (EC_50_s of ∼10 μM). Capsaicin and anandamide (AEA) activated TRPV1 receptors with an EC_50_ of 61 nM and 4.3 μM, respectively, but TRPV1 showed no response to Δ^9^-THC, cannabidiol, and other minor cannabinoids. Terpenoids did not significantly affect the responses of TRPA1 and TRPV1 receptors to submaximal and maximal concentrations of CA and Δ^9^-THC or the endocannabinoids AEA and 2-AG.

**Discussion:** We could not find any evidence that the terpenoids tested here activate TRPA1 and TRPV1 channels or modulate their activation by Δ^9^-THC and other agonists, including endocannabinoids.

## Introduction

*Cannabis* plant matter contains hundreds of diverse bioactive molecules. The major components of cannabis include phytocannabinoids such as the main psychoactive constituent Δ^9^-tetrahydrocannabinol (Δ^9^-THC) and the nonpsychoactive cannabidiol (CBD).^1^ However, cannabis contains other phytochemicals, including various minor phytocannabinoids, as well as flavonoid and terpenoid molecules, which have their own biological activities.^1,2^ The burgeoning utilization of medicinal cannabis necessitates research that addresses the complex polypharmacology of cannabis. Relevant to such research is the “entourage effect” hypothesis, which posits that the effects of the whole of the cannabis plant is substantially greater than the sum of its individual components. The “entourage effect” is often quoted by the growing community of cannabis entrepreneurs and patient advocates to explain the therapeutic and safety benefits of medicinal cannabis.

While few scientific studies have directly addressed the “entourage effect” hypothesis, there is accumulating evidence for cannabis plant extracts having effects that cannot simply be attributed to individual cannabis constituents.^3^ One early study showed that Δ^9^-THC-rich cannabis extracts had more potent effects than Δ^9^-THC administered as a purified substance in humans and animals, implying that other components in the extract might enhance the effects of Δ^9^-THC.^4^ Consistent with this finding more recent studies showed that Δ^9^-THC-rich cannabis extracts had greater anticancer effects than purified Δ^9^-THC on human cancer cells *in vitro* and *in vivo*.^5,6^

One means through which “entourage effects” may occur is via the interaction of individual components of the cannabis plant. Interplay between different cannabinoids provides one possibility, and numerous studies have examined interactions between the phytocannabinoids CBD and Δ^9^-THC. These studies have revealed great complexity in the interaction, with CBD synergistically enhancing or inhibiting the effects of Δ^9^-THC dependent on the measured variable and the frequency of dosing.^7–10^ It has also been hypothesised that the varying effects of different cannabis chemovars might be due to variation in their terpene content, which can reach concentrations of 3.5% and 10% in flower and trichomes, respectively.^11–13^ However, few studies have addressed this hypothesis; thus, the present study sought to advance the evidence base on potential terpenoid–cannabinoid interactions.

One means to deconvolute the complex actions of whole plant cannabis formulations on human biology is to assess whether phytochemical synergy occurs at the level of drug targets heterologously expressed in mammalian cells. We recently used this approach by examining whether several terpenoids influenced the agonist activity of Δ^9^-THC at cannabinoid 1 (CB_1_) and 2 (CB_2_) receptors expressed in AtT20 cells.^14^ The terpenoids analyzed failed to affect the actions of Δ^9^-THC on these receptors; however, this doesn't preclude interactions at other cannabinoid drug targets. In the present study we aimed to extend on our prior research by examining whether the terpenoids modulate the effects of phytocannabinoids at transient receptor potential ankyrin 1 (TRPA1) and transient receptor potential vanilloid 1 (TRPV1) channels.

TRPA1 channels play an important role in pain, itch, allergic cough, and asthma,^15^ and TRPV1 channels are involved in epilepsy, pain, thermoregulation, and itch.^15^ Phytocannabinoids are known agonists of these receptors, with Δ^9^-THC activating TRPA1, as well as CBD and other minor phytocannabinoids being reported to activate TRPV1.^16^ Furthermore, endocannabinoids affect these receptors, and anandamide (AEA) and 2-arachidonoylglycerol (2-AG) are TRPV1 and TRPA1 agonists, respectively.^17–19^ Given that TRPA1 and TRPV1 receptors appear to have allosteric sites, it is possible that entourage effects are mediated by terpenoid allosteric modulation of cannabinoid actions at these receptors.^20–22^ In the present study we aimed to assess whether seven terpenoids most commonly found in cannabis have activity at TRPV1 and TRPA1 channels alone, as well as observing whether they modulate the effects of phytocannabinoids and endocannabinoids on these receptors.

## Materials and Methods

### Cell culture

For this study, we used HEK293 cells as an expression system as they have been used extensively in previous studies assessing TRPV1 and TRPA1 channel signaling.^23–25^ Flp-In T-Rex HEK 293 cells (Life Technologies, Mulgrave, Victoria, Australia) were stably transfected with human TRPA1 (hTRPA1) or human TRPV1 (hTRPV1) cDNA (GenScript, Piscataway, NJ) and cultivated in Dulbecco's modified Eagle's medium supplemented with 10% fetal bovine serum (FBS), 100 U mL^−1^ penicillin, and 100 μg mL^−1^ streptomycin. Medium for transfected cells was supplemented with hygromycin B 80 μg mL^−1^ and blasticidin S 10 μg mL^−1^. Medium for Flp-In T-Rex HEK293 cells was supplemented with zeocin 100 μg mL^−1^ and blasticidin S 15 μg mL^−1^.

Cells were incubated in 5% CO_2_ at 37°C in a humidified atmosphere. Cells were grown in flasks with a surface area of 75 mm^2^, once at optimum confluence (∼90%); cells were trypsinized and seeded into a poly-D-lysine coated black, clear-bottomed 96-well plate (Corning, Castle Hill, NSW, Australia) at a density of 110,000–130,000 cells per well in L15 medium supplemented with 1% FBS, 15 mM glucose, 100 U penicillin, and 100 μg mL^−1^ streptomycin. The cells were plated in a volume of 80 μL and were incubated overnight in a humidified chamber at 37°C and ambient CO_2_. Expression of the hTRPA1 and hTRPV1 channel was induced 4.5 h before experimentation by addition of 2 μg mL^−1^ tetracycline per well.

### Calcium assay

Intracellular calcium [Ca]_i_ was measured using the Calcium 5 Kit from Molecular Devices (Sunnyvale, CA) and a Flexstation 3 microplate reader (Molecular Devices). Eighty microliters of dye dissolved in HEPES-buffered saline solution (HBSS) containing (in mM): NaCl 140, KCl 5.33, CaCl_2_ 1.3, MgCl_2_ 0.5, HEPES 22, Na_2_HPO_4_ 0.338, NaHCO_3_ 4.17, KH_2_PO_4_ 0.44, MgSO_4_ 0.4, glucose 10 (pH to 7.4) containing probenecid was loaded into each well of the plate for 1–1.5 h before testing in the Flexstation at 37°C. Fluorescence was measured every 2 sec (*λ*_excitation_=485 nm, *λ*_emission_=525 nm) for the duration of the experiment. Drugs were added after 2 min of baseline recording.

### Drug dilutions

All terpenoids, cinnamaldehyde (CA), capsaicin (CAPS), and Δ^9^-THC were made up in dimethyl sulfoxide (DMSO) and stored at −25°C. Protease activated receptor 1 (PAR-1) peptide agonist stock solution was made up in water and stored at −25°C. AEA and 2-AG stock solution were made up in DMSO and stored at −80°C. Fresh aliquots were used each day, and drugs were diluted in HBSS containing 0.01% bovine serum albumin immediately before the assay. The final concentration of DMSO per well did not exceed 0.2%. In each column of the 96-well plate, one blank addition well was included, where HBSS plus solvent was added to the cells. The changes in fluorescence produced by this blank addition were subtracted from drug responses before determining maximum change in [Ca]_i_ after each drug exposure.

### Drugs and reagents

CA, bovine serum albumin, 2-AG, and AEA were from Sigma-Aldrich (Castle Hill, NSW, Australia). PAR-1 peptide agonist was from AusPep (Tullamarine, VIC, Australia). Δ^9^-THC was purchased from THC Pharm GmbH (Frankfurt, Germany). Terpenoids were purchased from Sigma-Aldrich; (+)-α-pinene, (+)-β-pinene, (−)-β-caryophyllene (BCP), (±)-linalool, (R)-(+)-limonene, β-myrcene, and α-humulene. The purity of terpenoids as stated by the supplier was as follows: 98.5% ((+)-α-pinene, (+)-β-pinene), 98% ((−)-BCP), 97% ((±)-linalool, (R)-(+)-limonene), 93% (β-myrcene), and 96% (α -humulene). All tissue culture reagents were from Sigma-Aldrich, Life Technologies (Mulgrave, Victoria, Australia) or InvivoGen (San Diego, CA).

### Data analysis

Statistical analysis was performed using GraphPad Prism (Version 7.0b, San Diego, CA). The response to agonists was calculated as a percentage change of fluorescence over the baseline averaged for the 30 sec immediately before drug addition. All responses in HEK Flp-In T-Rex hTRPA1 cells were normalized to the response produced by a maximally effective concentration of CA (300 μM) included in each experiment. Responses in HEK Flp-In T-Rex hTRPV1 cells were normalized to the response produced by a maximally effective concentration of CAPS (10 μM) included in each experiment. Each determination was composed of two technical replicates, and the average of these replicates was used for analysis. Data for the response of hTRPA1 and hTRPV1 to terpenoids were extracted from experiments conducted to assess the effects of terpenoids on agonist-induced hTRPA1 and hTRPV1 activation, respectively, by analyzing the maximum change in [Ca]_i_ before 2-AG, CAPS, CA, AEA, or Δ^9^-THC application.

Concentration–response data were fit to a four-parameter logistic Hill equation to derive the EC_50_ values. Data are expressed as mean with standard error of the mean (SEM) of at least five independent experiments. Data were scrutinized for outliers using the ROUT method (*Q*=1%).^26^ For experiments assessing activation of either wild-type (WT), hTRPA1, or hTRPV1 cells by terpenoids and phytocannabinoids, unpaired *t*-tests were used to compare means. For all other experiments, means were compared using two-way analysis of variance (ANOVA) with Bonferroni's *post hoc* analysis. Experiments assessing the effects of terpenoids on CA-induced hTRPA1 activation and on CAPS-induced hTRPV1 activation were analyzed using two-way ANOVA with Bonferroni's *post hoc* on raw data expressed as percentage change in fluorescence. The null hypothesis was rejected if the *p*-value was lower than 0.05 (*p*>0.05=not significant).

## Results

### Terpenoids do not induce nonspecific intracellular Ca^2+^ mobilization in WT cells

We first wished to ensure that the terpenoids did not have nonspecific effects on intracellular Ca^2+^ increase in nontransfected HEK Flp-In T-Rex WT cells. WT cells were challenged with 10 μM of α-pinene, β-pinene, BCP, linalool, limonene, β-myrcene, or α-humulene (Supplementary Fig. S1A). WT cells endogenously express PAR-1,^27^ and their activation by a ligand induces intracellular Ca^2+^ mobilization.^28^ Hence, the application of 10 μM of the peptide agonist of PAR-1 was used as a positive control for the responsiveness of WT cells (Supplementary Fig. S1B, open circles). Supplementary Figure S1B shows that terpenoids at 10 μM did not change [Ca]_i_ in WT cells, ruling out confounding nonspecific effects of the terpenoids in experiments on hTRPA1- and hTRPV1-transfected cells.

### Terpenoids do not have agonist activity at hTRPA1 and do not modulate the agonist effects of CA, Δ^9^-THC, or 2-AG at hTRPA1

**FIG. 1.**
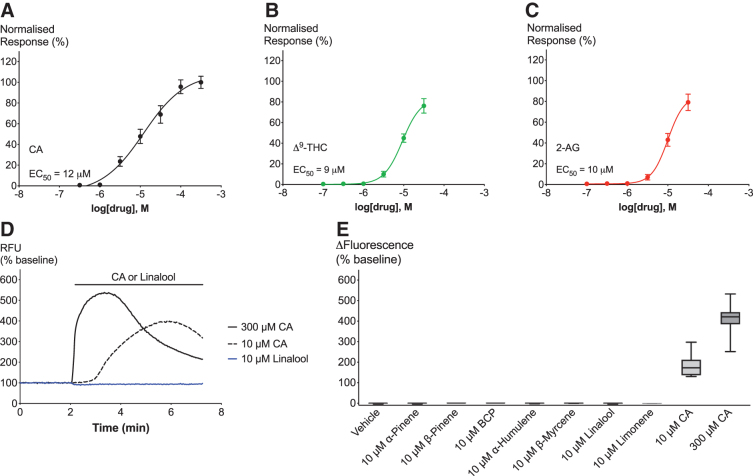
Effects of TRPA1 agonists and terpenoids on hTRPA1 cells. **(A–C)**. Concentration–response curves of hTRPA1 agonists CA, Δ^9^-THC, and 2-AG. **(D)** Representative trace of effects of 10 μM linalool (blue), 10 μM CA (black broken), and 300 μM CA (black full) on hTRPA1. **(E)** Maximum change in fluorescence from baseline upon application of vehicle, 10 μM terpenoid, 10 μM CA, or 300 μM CA. Data derived from Δ^9^-THC, CA, and 2-AG experiments (see Figs. 2–4). *N*>5, mean±SEM. Drugs were added for the duration of the bar. Δ^9^-THC, Δ^9^-tetrahydrocannabinol; 2-AG, 2-arachidonoylglycerol; CA, cinnamaldehyde; BCP, β-caryophyllene; hTRPA1, human transient receptor potential ankyrin 1; SEM, standard error of the mean.

hTRPA1 activation was determined by measuring maximum change of [Ca]_i_ in HEK Flp-In T-Rex cells expressing hTRPA1 channels. For the purpose of validating our hTRPA1 expressing cell system, we first analyzed the concentration–response relationship of an endogenous hTRPA1 agonist (2-AG), an exogenous agonist CA, and a phytocannabinoid agonist (Δ^9^-THC). Figure 1A–C shows the concentration response curves of all three ligands, which display an EC_50_ of 12 μM (CA), 9 μM (Δ^9^-THC), and 10 μM (2-AG). Calculation of the EC_50_ for Δ^9^-THC and 2-AG is restricted to changes in [Ca]_i_ evoked by concentrations of up to 30 μM, due to limited drug solubility. *E*_max_ of 30 μM agonist reached 76% (Δ^9^-THC) and 79% (2-AG), respectively, when normalized to the response of 300 μM CA. Neither CA, Δ^9^-THC, nor 2-AG showed a response in WT cells, which confirms their specificity for TRPA1 (Supplementary Fig. S1C).

**FIG. 2.**
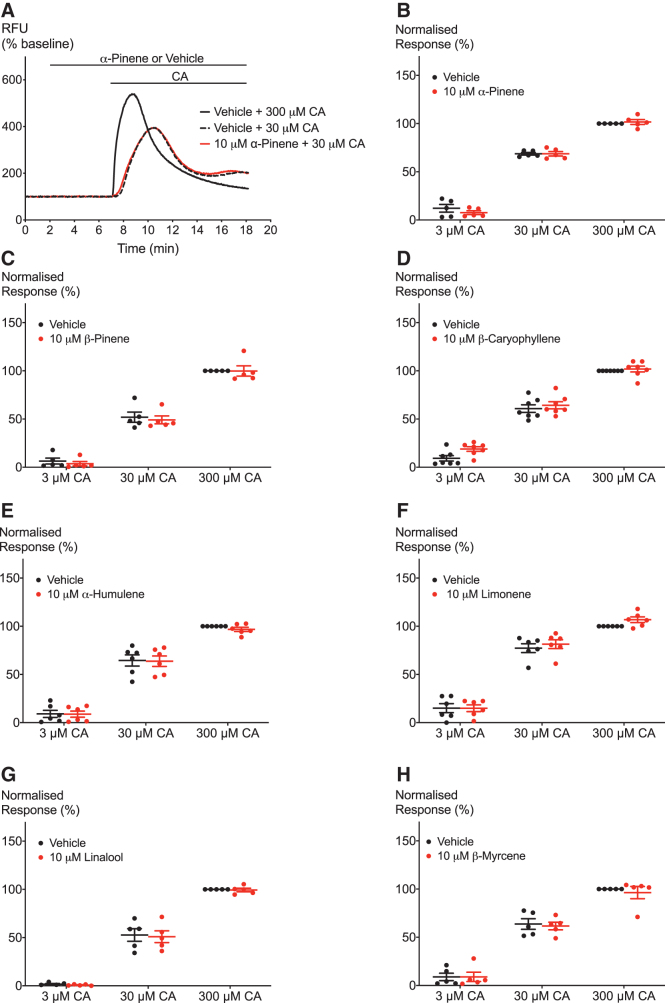
Effect of terpenoids on CA-induced hTRPA1 activation. **(A)** Representative trace showing the effect of preincubation with 10 μM α-pinene on activation of hTRPA1 by CA. **(B–H)** Terpenoids do not affect the activation of hTRPA1 by one maximal and two submaximal concentrations of CA. Two-way ANOVA with Bonferroni's *post hoc* analysis performed on raw data expressed as % change fluorescence, *N*=5–7, mean±SEM. Data presented as % of maximum CA (300 μM) response. Drugs were added for the duration of the bar. ANOVA, analysis of variance.

Following the establishment of hTRPA1 functional activation to known agonists in our cell system, we then sought to examine whether terpenoids commonly found in *Cannabis* activate hTRPA1 receptors. Considering the potency of known ligands at TRPA1, which show EC_50_ responses of around 10 μM (Fig. 1A–C), we decided to test 10 μM of α-pinene, β-pinene, BCP, linalool, limonene, β-myrcene, or α-humulene on hTRPA1 (Fig. 1D). We found no effect on [Ca]_i_ by any of the terpenoids (Fig. 1E). As expected, 10 μM and 300 μM CA showed a substantial increase in [Ca]_i_ (Fig. 1E).

**FIG. 3.**
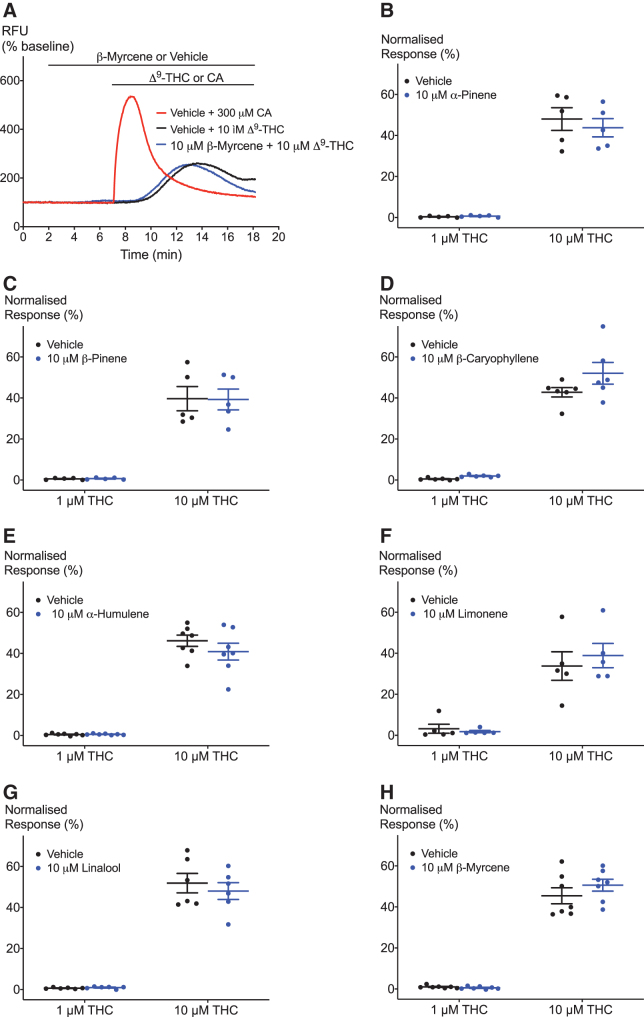
Effect of terpenoids on Δ^9^-THC-induced hTRPA1 activation. **(A)** Representative trace of effects of β-myrcene on hTRPA1 activation by 10 μM Δ^9^-THC. **(B–H)** Terpenoids do not affect the response of hTRPA1 to two submaximal Δ^9^-THC concentrations. Two-way ANOVA with Bonferroni's *post hoc* analysis, *N*=5–7, mean±SEM. Data presented as % of maximum CA (300 μM) response. Drugs were added for the duration of the bar.

**FIG. 4.**
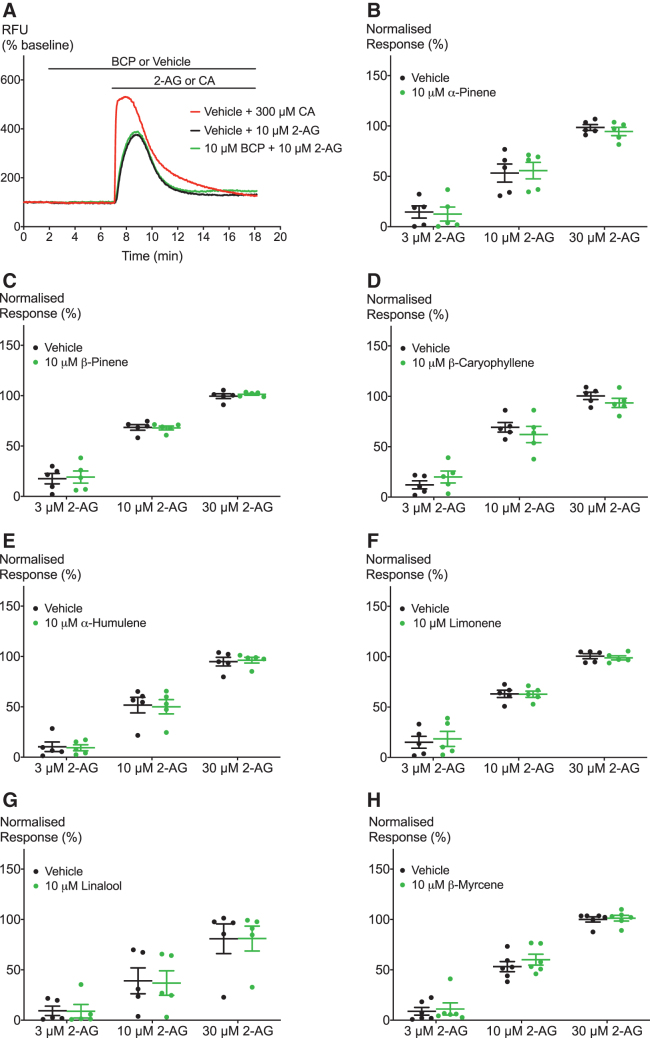
Effects of terpenoids on 2-AG-induced hTRPA1 activation. **(A)** Representative trace of effects of BCP on hTRPA1 activation by 10 μM 2-AG. **(B–H)** Terpenoids do not significantly modulate 2-AG-induced hTRPA1 signaling. Two-way ANOVA with Bonferroni's *post hoc* analysis, *N*=5–6, mean±SEM. Data presented as % of maximum CA (300 μM) response. Drugs were added for the duration of the bar.

The second aim was to investigate whether terpenoids have a modulatory effect on agonist-induced TRPA1 activation. To address this question, the response of hTRPA1 to a range of concentrations of the agonists CA, Δ^9^-THC, and 2-AG was measured after 5 min of application of 10 μM of terpenoid (Figs. 2–4A). Figures 2–4 show that none of the seven terpenoids tested significantly altered activation of hTRPA1 by any of the agonists. One limitation of working with the FLIPR Calcium assay is the fact that compounds cannot be removed from the solution. This might lead to continued activation of the channels and therewith a fluctuation in fluorescence over a prolonged period of time as seen in Figures 2A and 3A. Moreover, prolonged exposure to agonists can also lead to cytotoxicity as described by Stueber et al.^29^

### Terpenoids do not affect [Ca]_i_ through hTRPV1 and do not modulate hTRPV1 activation

**FIG. 5.**
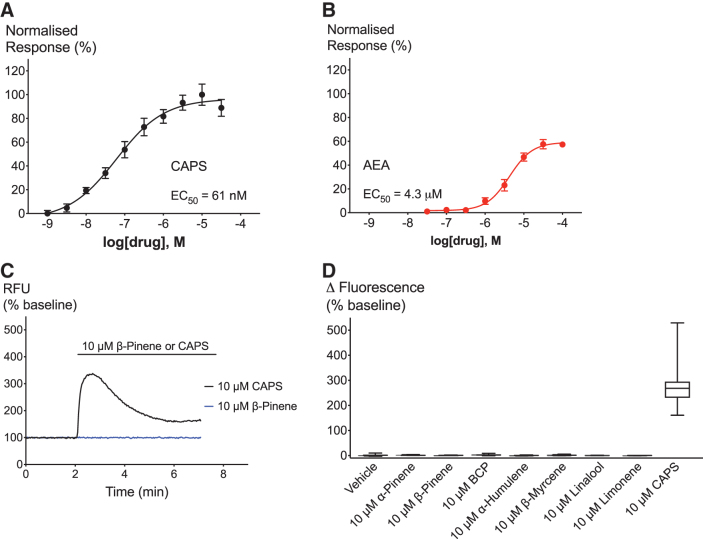
Effects of TRPV1 agonists and terpenoids on hTRPV1 cells. **(A, B)** Concentration–response curves of hTRPV1 agonists CAPS and AEA. **(C)** Representative trace showing the application of 10 μM β-pinene (blue) and 10 μM CAPS on hTRPV1 (black). **(D)** Maximum change in fluorescence upon application of vehicle, 10 μM terpenoid or 10 μM CAPS. *N*>5, mean±SEM, unpaired *t*-test. Drugs were added for the duration of the bar. AEA, anandamide; CAPS, capsaicin; hTRPV1, human transient receptor potential vanilloid 1.

For the experimental design and execution on HEK FlpIn T-Rex cells expressing hTRPV1 channels, we adopted a similar rational to the experiments performed on hTRPA1 cells. We first sought to validate our hTRPV1 channel-expressing cell system (hTRPV1) by determining the concentration–response curves of known agonists. Both the exogenous agonist CAPS and the endogenous agonist AEA activated hTRPV1 in a concentration-dependent manner with an EC_50_ of 61 nM for CAPS and 4.3 μM for AEA (Fig. 5A, B). The *E*_max_ of 100 μM AEA reached 57% when normalized to the response of 10 μM CAPS. Neither CAPS nor AEA showed a response in WT cells, which confirms their specificity for TRPV1 (Supplementary Fig. S1C). Surprisingly, we also saw no specific response to any of the common phytocannabinoids present in *Cannabis sativa* in hTRPV1 cells (Supplementary Fig. S2). Responses to 30 μM of cannabichromene, CBD, cannabidiolic acid, cannabigerol, cannabigerolic acid, cannabigerovarin, Δ^9^-THC, or tetrahydrocannabinolic acid were not significantly different between hTRPV1 (Supplementary Fig. S2A) and WT cells (Supplementary Fig. S2B).

We then sought out to determine the effects of terpenoids on hTRPV1 (Fig. 5C). hTRPV1 cells displayed significant response to 10 μM CAPS, but we saw no significant difference of the response to any of the seven terpenoids compared to vehicle (Fig. 5D).

**FIG. 6.**
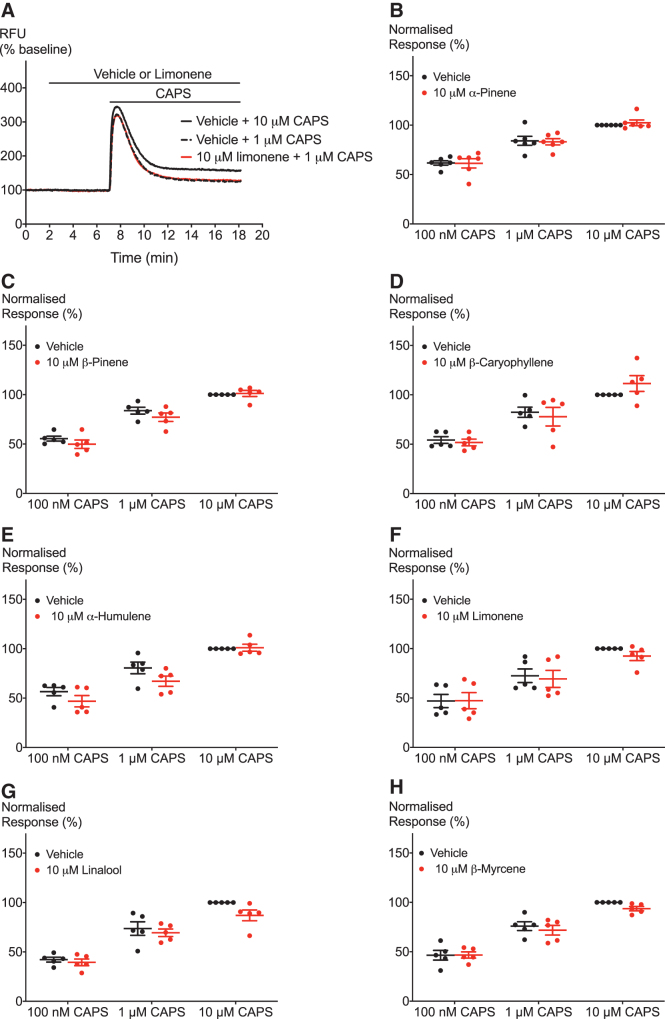
Effect of terpenoids on CAPS-induced hTRPV1 signaling. **(A)** Representative trace of effects of 10 μM limonene on TRPV1 activation by 1 μM CAPS. **(B–H)** Terpenoids do not significantly change hTRPV1 response to one maximal and two submaximal concentrations of CAPS. Two-way ANOVA with Bonferroni's *post hoc* analysis performed on raw data expressed as % change fluorescence, *N*=5, mean±SEM. Data presented as % of maximum CAPS (10 μM) response. Drugs were added for the duration of the bar.

**FIG. 7.**
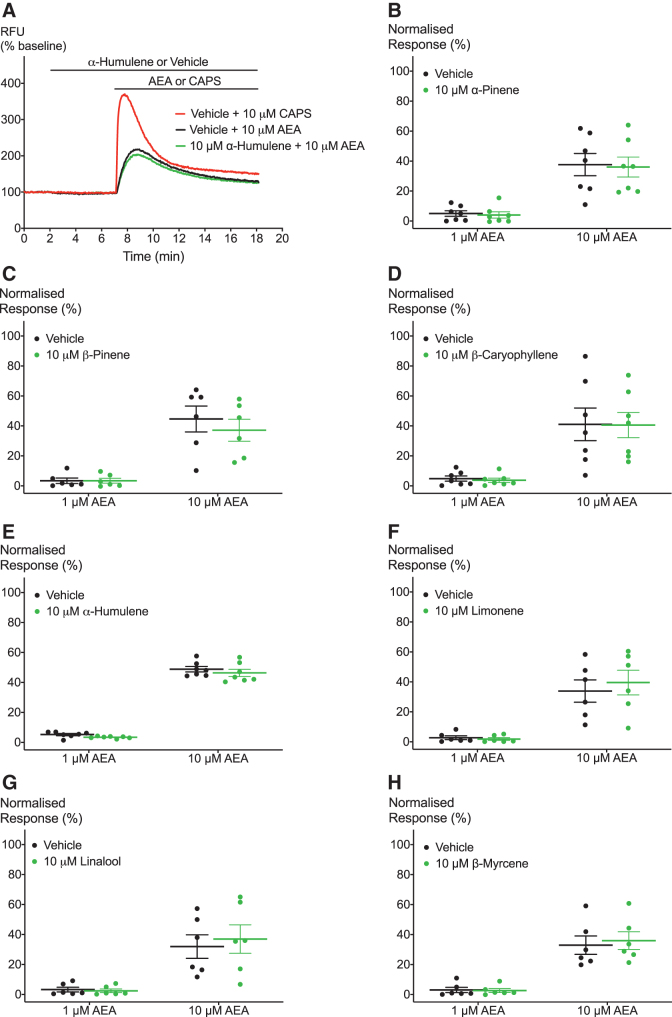
Effects of terpenoids on hTRPV1 activation by AEA. **(A)** Representative trace of effects of 10 μM α-humulene on hTRPV1 activation by 10 μM AEA. **(B–H)** Application of terpenoids does not lead to significant change in hTRPV1 activation by AEA at two submaximal concentrations. Two-way ANOVA with Bonferroni's *post hoc* analysis. *N*=5–7, mean±SEM. Data presented as % of maximum CAPS (10 μM) response. Drugs were added for the duration of the bar.

Finally, we aimed to examine the effects of terpenoids on hTRPV1 activation by known agonists. Again, we tested the effects of 10 μM of the terpenoids on activation of hTRPV1 by an exogenous agonist CAPS and an endogenous agonist AEA. We applied 10 μM of the terpenoids for 5 min followed by addition of a range of concentrations of the agonist (Figs. 6A and 7A). Figures 6 and 7 show that none of the seven terpenoids significantly shifted the response of hTRPV1 to CAPS and AEA, respectively.

Since we did not see a response of hTRPV1 to any of the common phytocannabinoids (Supplementary Fig. S2), we were unable to study the effects of terpenoids on the activation of hTRPV1 by other phytocannabinoids.

## Discussion

Our work was inspired by the idea that terpenoids might influence the actions of endocannabinoids and phytocannabinoids at their receptor targets.^11,30^ Terpenoid-cannabinoid synergy at cannabinoid drug targets may provide one mechanism for the “entourage effect:” the notion that the pharmacological effects of the cannabis plant, and its mixture of 500 bioactive molecules, are greater than the sum of its individual components. We have addressed this hypothesis recently by examining the interaction between terpenoids and Δ^9^-THC at CB_1_ and CB_2_ receptors but could not find any evidence of terpenoid modulation of the actions of Δ^9^-THC at these receptors.^14^ In this study we further explored the entourage hypothesis by examining potential interactions at TRPV1 and TRPA1 channels. The principal findings of our work are that seven terpenoids commonly found in *Cannabis*, namely α-pinene, β-pinene, BCP, linalool, limonene, β-myrcene, and α-humulene, did not elicit a change in [Ca]_i_ in either hTRPA1 or hTRPV1 expressing cells and did not affect activation of either receptor by endocannabinoids and the phytocannabinoid Δ^9^-THC.

The validation of our cell system makes us confident that they provide reliable tools to study hTRPA1 and hTRPV1 functional activation. Our data on the potency of CA (EC_50_=12 μM) and Δ^9^-THC (EC_50_=9 μM) to activate TRPA1 channels are consistent with the literature.^19,24,31^ We report here for the first time that 2-AG is a TRPA1 receptor agonist. Interestingly, 2-AG had an almost identical concentration–response curve to Δ^9^-THC with a similar EC_50_ (10 μM) and *E*_max_. This finding is consistent with research showing that endocannabinoids activate TRP channels.^32^ We also showed that our TRPV1 cell system is sensitive to the effects of known agonists. CAPS and AEA activated hTRPV1 with comparable potency to prior reports (EC_50_=61 nM and 4.3 μM, respectively).^17,19,33^ None of the agonists changed [Ca]_i_ in our WT cells confirming the specificity of these agonists.

To date, only two studies have demonstrated an effect of terpenoids from *Cannabis* on TRPA1 and TRPV1.^34,35^ Riera et al. showed that linalool activated human TRPA1 but only at very high concentrations (EC_50_ of 117 μM).^34^ Consistent with our results here, Riera et al. showed that linalool had no effect on TRPV1.^34^ Our results confirmed the findings of Jansen and coworkers who showed that BCP, limonene, linalool, β-pinene, and α-humulene do not activate rat TRPV1.^35^ Yet, unlike the null finding reported here with myrcene against human TRPV1, they showed that 10 μM β-myrcene activated rat TRPV1 when using the Fluo-4 calcium assay to assess changes in [Ca]_i_. We did not see a significant or specific response of hTRPV1 to any of the phytocannabinoids commonly found in *Cannabis* and hence did not address the potential of terpenoids to modulate CBD-induced TRPV1 activation.

The pharmacological validation of our cell systems presented herein demonstrates clearly that our cells are sensitive to known agonists of TRPA1 and TRPV1. However, that the phytocannabinoids failed to activate hTRPV1 here is contrary to the existing literature.^16,25,35,36^ For example, CBD has been shown to be a TRPV1 channel agonist with potencies between 1 and 30 μM, but in our study CBD did not activate hTRPV1 cells at a 30 μM concentration.^16,25^ There are numerous differences in assay conditions that may help explain the discrepant findings, including differences in expression levels of TRPV1, which may render other systems more sensitive to the effects of cannabinoids.^16,25,35,36^ Differences in the method utilized to measure changes in intracellular calcium, the temperature at which the experiments were performed, and the concentration of solvents might also contribute to the discordant findings.^37–39^

The present study is limited to assessing terpenoid and cannabinoid interactions in a HEK cell tetracycline-regulated expression system. Future studies could use whole cell patch clamp electrophysiology in brain slices to assess effects on TRPA1 and TRPV1 channels in a native system that better mimics *in vivo* complexity.^40^ Moreover, the translational potential of our results might be improved by utilizing neurons derived from human inducible pluripotent stem cells, which have been developed to study TRP channel responses.^41^ Another limitation of the current study is that we have only studied the seven terpenoids that are most commonly reported to be present in *Cannabis*; however, there are likely to be hundreds of these molecules in the plant.^2^ With increasing knowledge of the terpenoid content of cannabis, novel terpenoid molecules will need to be incorporated in studies assessing terpenoid–cannabinoid interactions.

Collectively our studies show that there is currently no evidence for terpenoid–cannabinoid interactions at CB1, CB2, TRPV1, or TRPA1 receptors, but given the promiscuity of the cannabinoids, the search should continue by exploring interactions at additional molecular targets.^14^ For example, terpenoid modulation of Δ^9^-THC effects at TRPV2, TRPV3, GPR18, GPR55, glycine, and PPARγ receptors could be examined.^42^ Moreover, *in vivo* research is required as synergy might be attained through effects on distinct but intersecting biochemical systems.^43^ Pharmacokinetic entourage might play a role where the terpenoids modulate the absorption, distribution, metabolism, and excretion of the cannabinoids. The complexity of both human biochemistry and cannabis phytochemistry demands that mechanisms explaining possible entourage effects will be multifaceted. Increased global utilization of medicinal cannabis necessitates research on the entourage effect to continue in earnest.

## Supplementary Material

Supplemental data

Supplemental data

## Abbreviations Used

Δ^9^-THC: Δ^9^-tetrahydrocannabinol
2-AG: 2-arachidonoylglycerol
AEA: anandamide
ANOVA: analysis of variance
BCP: β-caryophyllene
CA: cinnamaldehyde
CAPS: capsaicin
CBD: cannabidiol
DMSO: dimethyl sulfoxide
FBS: fetal bovine serum
HBSS: HEPES-buffered saline solution
hTRPA1: human transient receptor potential ankyrin 1
hTRPV1: human transient receptor potential vanilloid 1
PAR-1: protease activated receptor 1
SEM: standard error of the mean
WT: wild-type
