# Supplementary material for: Terpenoids Commonly Found in Cannabis sativa Do Not Modulate the Actions of Phytocannabinoids or Endocannabinoids on TRPA1 and TRPV1 Channels
Source: Cannabis Cannabinoid Res. 2020 Dec 15;5(4):305–17. doi: 10.1089/can.2019.0099 (PMC7759271; doi:10.1089/can.2019.0099)
Supplement: Supplemental data [file Supp_FigS1.pdf]

Supplementary Data

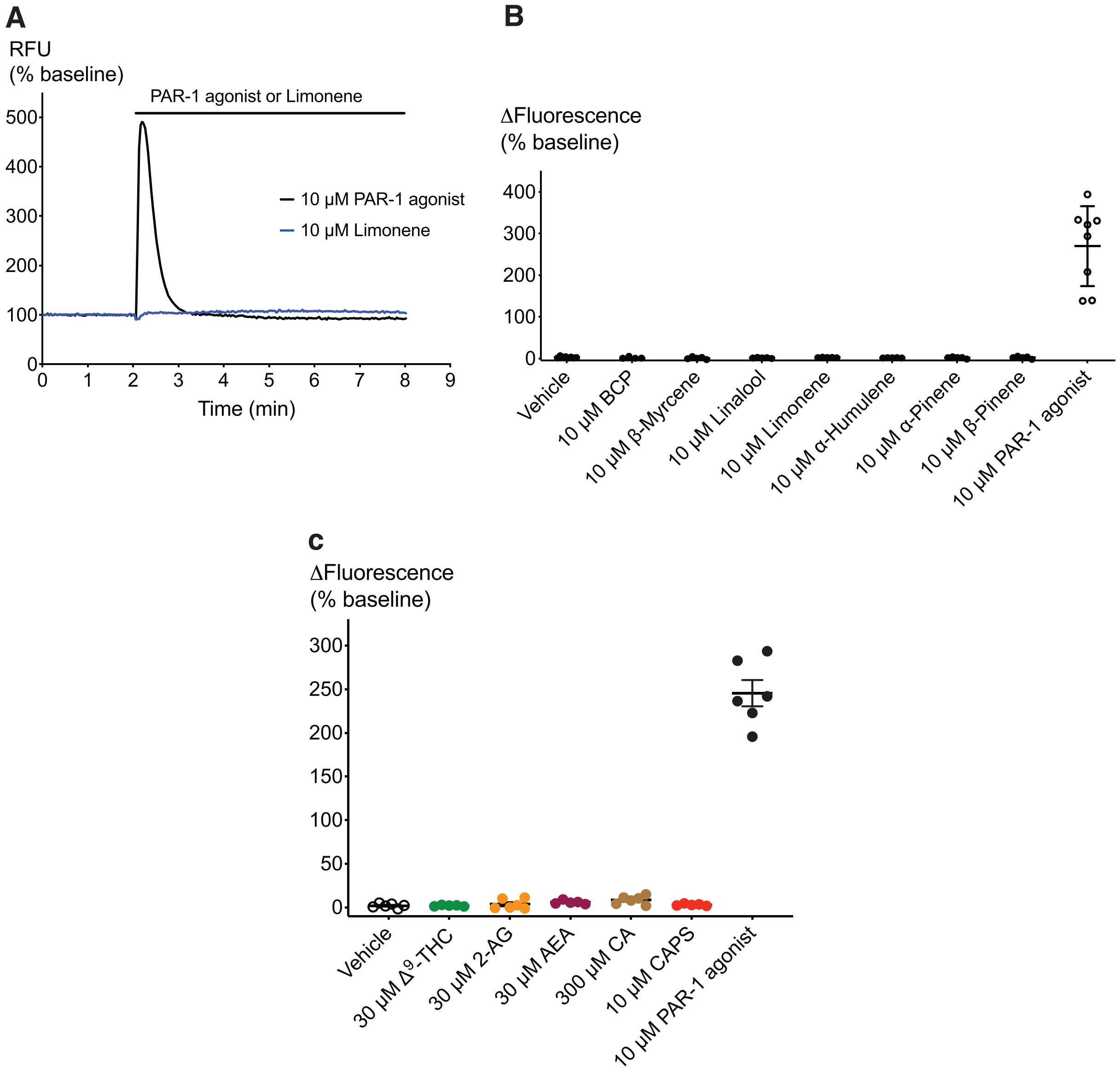

SUPPLEMENTARY FIG. S1. WT cells do not respond to terpenoids and TRPA1 and TRPV1 agonists. **(A)** Representative trace showing the effects of 10 μM limonene (blue) and 10 μM PAR-1 agonist (black) on WT cells. **(B)** Maximum change in fluorescence from baseline upon application of 10 μM terpenoid or 10 μM PAR-1 agonist. **(C)** CAPS, Δ^9-THC, AEA, 2-AG, and CA do not induce changes in $[Ca]_i$ in WT cells. Unpaired *t*-test. $N = 5$–8, mean ± SEM. Drugs were added for the duration of the bar. Δ^9-THC, Δ^9-tetrahydrocannabinol; 2-AG, 2-arachidonoylglycerol; AEA, anandamide; CA, cinnamaldehyde; CAPS, capsaicin; PAR-1, protease activated receptor 1; SEM, standard error of the mean; TRPA1, transient receptor potential ankyrin 1; TRPV1, transient receptor potential vanilloid 1; WT, wild-type.
