# Supplementary material for: Terpenoids Commonly Found in Cannabis sativa Do Not Modulate the Actions of Phytocannabinoids or Endocannabinoids on TRPA1 and TRPV1 Channels
Source: Cannabis Cannabinoid Res. 2020 Dec 15;5(4):305–17. doi: 10.1089/can.2019.0099 (PMC7759271; doi:10.1089/can.2019.0099)
Supplement: Supplemental data [file Supp_FigS2.pdf]

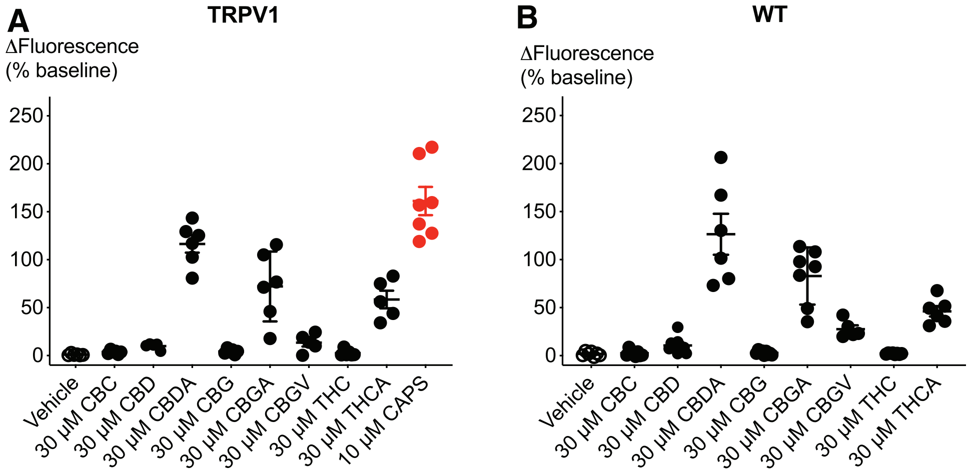

SUPPLEMENTARY FIG. S2. Effects of phytocannabinoids on hTRPV1 and WT cells. **(A)** Response of hTRPV1 cells to common phytocannabinoids. **(B)** CBDA, CBGA, CBGV, and THCA induce an increase in $[Ca]_i$ in WT cells. Effects of phytocannabinoids are not significantly different between hTRPV1 and WT cells. Unpaired *t*-test. $N > 5$, mean ± SEM. CBDA, cannabidiolic acid; CBGA, cannabigerolic acid; CBGV, cannabigerovarin; hTRPA1, human transient receptor potential ankyrin 1; hTRPV1, human transient receptor potential vanilloid 1; THCA, tetrahydrocannabinolic acid.
